# Temporal Trend in Selective Cyclooxygenase‐2 Inhibitors Sales in Brazilian Drugstores

**DOI:** 10.1002/pds.70168

**Published:** 2025-06-07

**Authors:** Tayanny Margarida Menezes Almeida Biase, Marcus Tolentino Silva, Larissa Lopes, Taís Freire Galvao

**Affiliations:** ^1^ Faculdade de Ciências Farmacêuticas Universidade Estadual de Campinas São Paulo Brazil; ^2^ Departmento de Saúde Coletiva, Faculdade de Ciências da Saúde Universidade de Brasília Brasília Brazil

**Keywords:** Brazil, celecoxib, cyclooxygenase 2 inhibitors, drug utilization, etoricoxib, pharmacoepidemiology, time trend analysis

## Abstract

**Purpose:**

To assess the trends in selective cyclooxygenase‐2 inhibitor anti‐inflammatory drugs (coxibs) sales in Brazil from 2014 to 2021.

**Methods:**

A time trend analysis of coxibs sales in Brazil from January 2014 to December 2021 was conducted using the Brazilian National Controlled Products Management System. Primary outcomes consisted of coxibs sales in defined daily dose (DDD) and DDD per 1000 inhabitants per day (DID), analyzed by Brazilian region (North, Northeast, South, Southeast, and Midwest). The trends in coxib consumption were analyzed using a segmented regression model, and the average annual percent change (AAPC) with a 95% confidence interval (95% CI) was calculated.

**Results:**

Celecoxib and etoricoxib sales increased in Brazil from 2014 to 2021. Celecoxib sales rose from 0.2 to 0.4 DID (AAPC 15.0; 95% CI 8.9, 21.5), particularly in the South, from 0.4 in 2014 to 0.7 DID in 2021 (AAPC 11.8; 95% CI 6.9, 16.9). Etoricoxib sales increased from 0.1 to 0.2, especially in the Midwest (AAPC 10.6; 95% CI 5.5, 16.0). Northern Brazil presented the lowest coxibs' consumption, which also increased in the period. National changes in etoricoxib sales were observed between 2014 and 2018 (annual percent change, APC 12.2; 95% CI −2.0, 28.4) and 2018 and 2021 (APC −3.6; 95% CI −22.1, 19.3).

**Conclusion:**

Coxibs sales in Brazil increased from 2014 to 2021, and celecoxib was the most used coxib. Shift changes in sales were observed in some regions for both coxibs, and nationally for etoricoxib. Prescription retention requirements for coxibs sales, instituted a decade before this analysis, potentially did not reduce consumption.

**Summary**

- An increasing trend in coxibs sales was observed from 2014 to 2021 in Brazil, considering population growth in the period.
- The inclusion of coxibs in the Brazilian controlled drugs and substances act, which occurred in the decade prior to the analysis, does not appear to have affected consumption.
- Celecoxib had higher consumption in the Brazilian Southern and Midwestern regions. Etoricoxib had higher sales in the Midwest and South.
- Structural breaks were observed nationally in etoricoxib sales from 2014 to 2018 and from 2018 to 2021, indicating a change in sales trend countrywide.

## 1 Introduction

Anti‐inflammatory drugs are widely prescribed for various clinical indications, typically for limited durations in order to minimize potential harmful effects [1]. Selective cyclooxygenase‐2 inhibitor anti‐inflammatory drugs (coxibs) were introduced as a newer class of nonsteroidal anti‐inflammatory drugs with fewer adverse effects [2]. After their introduction to the market, initial evidence emerged regarding cardiovascular adverse events, including an increased risk of myocardial infarction, stroke, heart failure, and death [3, 4].

Regulatory agencies then have implemented measures to mitigate the risks associated with coxibs use [5, 6]. In the United States, the Food and Drug Administration mandated the inclusion of black box warnings on all coxibs packaging to alert prescribers and patients about the increased risk of cardiovascular events, resulting in the withdrawal of rofecoxib and valdecoxib due to safety concerns [5, 7]. Similarly, the European Medicines Agency drove the removal of rofecoxib, valdecoxib, and lumiracoxib from the European market [6]. In turn, etoricoxib, while approved in Europe, did not receive authorization in the United States, illustrating the diverse regulatory stances worldwide [8].

In Brazil, coxibs were initially available under medical prescription. Following the emergence of adverse cardiovascular events reported by different regulatory agencies, they were classified in 2008 as controlled drugs by the Brazilian National Health Surveillance Agency (*Agência Nacional de Vigilância Sanitária*, Anvisa), requiring retention of the medical prescription for sales of these medicines [9].

Following these initial regulatory measures, few studies have focused on investigating the risks associated with coxibs [10, 11]. Lesser known adverse reactions for this drug class, such as renal adverse effects, have been documented [12], but this higher risk seems to be overlooked by both prescribers and patients. Limited interest in further research on coxibs may lead to the neglect of other potential safety concerns, highlighting the need for proper pharmacovigilance and risk assessments to better monitor the population exposure to these drugs.

Nonsteroidal anti‐inflammatory drugs, including coxibs, represented the third most consumed drug in primary care in Brazil, in 2015 [13]. Specific studies on their consumption are scarce and, when available, generally do not distinguish coxibs from other anti‐inflammatory drugs [13, 14]. Absence of administrative databases that allow analyses based on sales records also limits evaluating the prescribing and dispensing patterns of drug utilization in the country. The Brazilian National Controlled Products Management System (*Sistema Nacional de Gerenciamento de Produtos Controlados*, SNGPC), established in 2007 by Anvisa to record sales of controlled drugs nationwide [15], embodies one of the few available sources for drug utilization studies, provided they are controlled drugs, such as the coxibs. In 2020, the SNGPC dataset has been made publicly available [16], resulting in analyses focused on psychotropic [17] drugs and antibiotics [18]. Investigating coxibs sales trends based on Brazilian drugstores sales records would allow a better understanding of their consumption and generate evidence to assess prescribing and dispensing patterns for these drugs. The aim of this study was to assess the trends in coxibs sales in Brazil from 2014 to 2021 and to investigate countrywide regional differences in coxib consumption.

## 2 Methods

### 2.1 Study Design

This is a time trend study of coxibs sales in Brazilian drugstores from January 2014 to December 2021.

### 2.2 Setting

We used data available in the SNGPC, which records sales of controlled drugs in Brazilian drugstores and compounding pharmacies, but does not cover hospitals and other health services [15].

Data collection spanned from 2014 to 2021, since in December 2021 Anvisa decreed a temporary suspension of obligatory data transmission to the SNGPC due to instabilities in this electronic system [19].

### 2.3 Participants

We considered eligible sales records from drugstores of celecoxib and etoricoxib. As parecoxib—another coxib licensed in Brazil—is a parenteral drug and only used in hospitals and clinics, it was not eligible for this study.

### 2.4 Variables

The primary outcome was coxibs sales in terms of defined daily dose (DDD) and DDD per 1000 inhabitants per day (DID). Independent variables included year of sale, Brazilian region (North, Northeast, South, Southeast, and Midwest), active ingredient (celecoxib, etoricoxib), commercial presentation of the drug and pharmaceutical form, and quantity of units.

### 2.5 Data Sources and Measurement

Data were obtained from the SNGPC dataset provided by Anvisa [16]. The amount of DDDs (average daily dose of treatment per drug for its main indication in adults) of each coxib was calculated using a two‐step process: first, the dosage of the active substance per package was multiplied by the number of units sold; second, this total was divided by the corresponding DDD assigned by the Anatomical Therapeutic Chemical (ATC) classification of the World Health Organization ATC/DDD Index [20]. The total DDD of each coxib was divided by the estimated population of each region and year [21], per day to obtain the DID (DDD per 1000 inhabitants per day) [20].

### 2.6 Statistical Methods

Initially, the variables were assessed descriptively, obtaining the absolute and relative frequencies of coxibs sales in Brazil, in DID, using Stata version 14.2 (StataCorp, College Station, TX). We removed individual records with sales of more than 17 000 units, which likely occurred due to typing errors in the SNGPC and were considered outliers in our analysis.

The trends in the consumption of anti‐inflammatory drugs in DID were analyzed using a segmented regression model (joinpoint regression), aggregated by year for region and country, to test whether a line with multiple segments—each with its own unique linear trend—better described temporal changes compared to a single linear trend [22]. The average annual percent change (AAPC) was calculated with a 95% confidence interval (95% CI), and statistical significance was tested by the chi‐square test, with *p* < 0.05 considered statistically significant. When structural breaks (joinpoints) were identified, separate linear trends were fitted between evaluations of trend changes over time, and the annual percent change (APC) was calculated for the related periods [22, 23]. These analyses were performed using the Joinpoint Regression Program, version 5.0.2 (Statistical Research and Applications Branch, National Cancer Institute).

## 3 Results

From 2014 to 2021, 3 386 363 coxibs sales were recorded, from which 15 records were considered outliers and six parecoxib sales were excluded.

**FIGURE 1:**
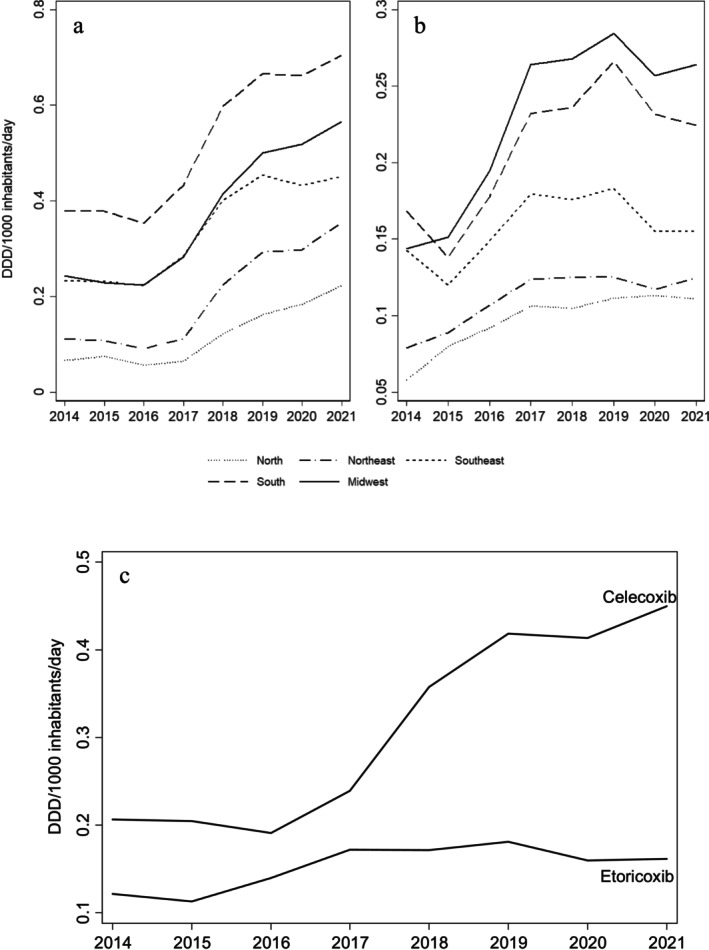
Trends in celecoxib (a) and etoricoxib (b) sales per region and nationally (c) in defined daily doses per 1000 inhabitants per day from 2014 to 2021.

Sales of both coxibs increased in the period assessed. Celecoxib had higher consumption, particularly in the South (0.4 DID to 0.7 DID) and Midwest (0.2 DID to 0.6 DID) (Figure 1a). Etoricoxib sales were highest in the Midwest (0.2 DID to 0.3 DID), followed by the South (0.2 DID to 0.2 DID) (Figure 1b). Northern Brazil consistently presented the lowest consumption records of both drugs. Coxibs sales noticeably increased from 2014 to 2021, with celecoxib ranking as the highest selling drug, with the highest growth (0.2 DID to 0.4 DID) than etoricoxib (0.1 DID to 0.2 DID) (Figure 1c).

Nationally, celecoxib sales increased from 0.2 DID to 0.4 DID and the trend in the annual change was significant (AAPC 15.0; 95% CI 8.9, 21.5; *p* < 0.001). Changes in sales were higher in the Northern (AAPC 19.5; 95% CI 1.8, 40.3; *p* = 0.029) and Midwest regions (AAPC 16.5; 95% CI 10.3, 23.0; *p* < 0.001). Between 2014 and 2016, structural breaks in celecoxib sales were identified in the Northern (APC –8.6; 95% CI −58.8, 102.5; *p* = 0.742) and Northeastern (APC –5.5; 95% CI −66.1, 163.5; *p* = 0.871) regions (Table 1).

**TABLE 1:** Sales of selective cyclooxygenase‐2 inhibitor anti‐inflammatory drugs (coxibs) in defined daily dose per 1000 inhabitants per day, average annual percent change (AAPC), and 95% confidence interval (95% CI) according to the Brazilian region from 2014 to 2021.

Region	2014	2015	2016	2017	2018	2019	2020	2021	AAPC (95% CI)	*p*
Celecoxib										
North	0.1	0.1	0.1	0.1	0.1	0.2	0.2	0.2	19.5 (1.8, 40.3)^a^	0.029
Northeast	0.1	0.1	0.1	0.1	0.2	0.2	0.3	0.4	20.6 (−2.0, 48.3)^b^	0.076
Southeast	0.2	0.2	0.2	0.3	0.4	0.5	0.4	0.5	13.0 (7.4, 18.8)	< 0.001
South	0.4	0.4	0.4	0.4	0.6	0.7	0.7	0.7	11.8 (6.9, 16.9)	< 0.001
Midwest	0.2	0.2	0.2	0.3	0.4	0.5	0.5	0.6	16.5 (10.3, 23.0)	< 0.001
Brazil	0.2	0.2	0.2	0.2	0.4	0.4	0.4	0.5	15.0 (8.9, 21.5)	< 0.001
Etoricoxib										
North	0.1	0.1	0.1	0.1	0.1	0.1	0.1	0.1	10.0 (7.7, 12.4)	< 0.001
Northeast	0.1	0.1	0.1	0.1	0.1	0.1	0.1	0.1	6.6 (3.8, 9.6)^c^	< 0.001
Southeast	0.1	0.1	0.2	0.2	0.2	0.2	0.2	0.2	2.6 (−6.8, 13.0)^d^	0.600
South	0.2	0.1	0.2	0.2	0.2	0.3	0.2	0.2	5.9 (−7.2, 20.8)^e^	0.397
Midwest	0.1	0.2	0.2	0.3	0.3	0.3	0.3	0.3	10.6 (5.5, 16.0)^f^	< 0.001
Brazil	0.1	0.1	0.1	0.2	0.2	0.2	0.2	0.2	5.1 (−2.4, 13.1)^g^	0.185

*Note:* The annual percent changes (APC) of structural breaks identified in each segment are as follows:

^a^ 2014–2016: −8.6 (95% CI −58.8, 102.5), *p* = 0.742; 2016–2021: 33.1 (95% CI 11.4, 59.0), *p* = 0.014.

^b^ 2014–2016: −5.5 (95% CI −66.1, 163.5), *p* = 0.871; 2016–2021: 33.0 (95% CI 5.7, 67.2), *p* = 0.028.

^c^ 2014–2017: 16.8 (95% CI 7.9, 26.4), *p* = 0.008; 2017–2021: −0.4 (95% CI −5.3, 4.8), *p* = 0.828.

^d^ 2014–2018: −9.1 (95% CI −8.5, 30.1), *p* = 0.213; 2018–2021: −5.5 (95% CI −28.45, 24.8), *p* = 0.565.

^e^ 2014–2019: 12.3 (95% CI −3.0, 30.0), *p* = 0.086; 2019–2021: −8.7 (95% CI −52.6, 75.8), *p* = 0.688.

^f^ 2014–2017: 24.8 (95% CI 8.8, 43.3), *p* = 0.014; 2017–2021: 1.0 (95% CI −7.4, 10.2), *p* = 0.735.

^g^ 2014–2018: 12.2 (95% CI −2.0, 28.4), *p* = 0.073; 2018–2021: −3.6 (95% CI −22.1, 19.3), *p* = 0.621.

Etoricoxib changed from 0.1 DID in 2014 to 0.2 DID in 2021, but the average yearly change trend was not significant (AAPC 5.1; 95% CI −2.4, 13.1; *p* = 0,185), with a structural break between 2014 and 2018 (APC 12.2; 95% CI –2.0, 28.4; *p* = 0.073) and 2018 and 2021 (APC –3.6; 95% CI –22.1, 19.3; *p* = 0.621). Regionally, structural breaks were identified in all regions except the Northern Brazil, which presented a greater annual change trend (AAPC 10.0; 95% CI 7.7, 12.4; *p* < 0.001), along with the Midwest (AAPC 10.6; 95% CI 5.5, 16.0; *p* < 0.001).

## 4 Discussion

From 2014 to 2021, the overall consumption of coxibs increased in Brazil. Celecoxib presented the highest growth, both at the national and regional levels. The consumption of etoricoxib also exhibited an increasing trend, which was not consistent and presented structural breaks nationally and in most Brazilian regions during this period, with higher consumption in the South and Midwest regions. The present findings considered population growth during the period and indicate a consistent increase in coxibs sales in Brazil.

Our study was based on SNGPC records, a reliable database on the use of controlled drugs by outpatients in Brazil that has important limitations such as allowing retroactive entries, which can increase bias [24]. The system requires prescriber identification (nonmandatory), but lacks essential demographic data to allow proper pharmacoepidemiological assessment, such as sex and age. Clinical indications are also unavailable in the system, which hinders assessing prescription appropriateness and other factors regarding medical practices and patient needs. We restricted our analysis to 2021 due to system instabilities that led Anvisa to temporarily suspend mandatory sales data reporting [19], and our data also do not cover drugs dispensed in compounding pharmacies and health services, which limits the representativeness of the results.

Parecoxib sales were not included in the analysis, since it is used only in the hospital setting and also limits our findings representativeness. A retrospective drug utilization study conducted in a hospital in Malaysia in 2018 revealed that among 195 postoperative patients who used nonsteroidal anti‐inflammatory drugs, 64% received a coxibs, and the parecoxib consumption rate was nearly four times higher, with a per‐year DDD of 759 and 389.2 DDDs per 100 admissions [2]. It preference in perioperative care is attributed to its specific mechanism, as it mitigates local inflammatory responses [25], better gastrointestinal safety [26], particularly in the elderly and pediatric surgical patients [27, 28].

Overall consumption of coxibs increased in Brazil in this 8‐year time trend and contradicted the trends observed in countries where coxibs prescriptions declined following regulatory restrictions [29, 30]. Our analysis did not cover the start of restriction of coxibs sales by including them in the controlled drugs and substances act in 2008, but it raises concerns whether the measures implemented in Brazil promoted rational use of these drugs, as planned. The increase in sales took into account population growth during the period, but our results were not adjusted for age. Aging may explain the increased use of these drugs, as it is a relevant factor in Brazilian demographic transition [31]. Despite this, the observed increase in coxib consumption in Brazil aligned with other settings. In a study in Nordic countries, etoricoxib consumption in Finland increased from 5.7 DID in 2010 to 6.7 DID in 2016 [32]. Denmark, Iceland, Norway, and Sweden had moderate rates of consumption after 2006 [32]. Another comparative analysis of several European countries highlighted a significant growth of 420% in etoricoxib consumption in Montenegro, from 0.05 DID in 2010 to 0.26 DID by 2019 [33]. A study conducted in China reported an increasing use of celecoxib in 2018, which rated among the top three most utilized analgesics, with a DID of 1.47 [34]. Several clinical guidelines recommend the use of coxibs for inflammatory diseases and pain management [35, 36]. Stricter regulations on coxibs safety and updates in clinical guidelines may have contributed to shifts in prescribing patterns, including the increased preference for coxibs in certain settings [37, 38, 39].

Trend shifts in celecoxib and etoricoxib sales were observed in Brazilian regions, and nationally for etoricoxib, indicating changes in coxib consumption over time. This study does not allow us to identify the specific causes behind these structural shifts. Several factors may contribute to changes in drug sales, including cost fluctuations [40], regulatory policies [41], prescribing pressures [42], and marketing strategies [43]. Studies that assessed nonsteroidal anti‐inflammatory drug utilization trends found that easy accessibility, broad medical indications, and the decline in opioid prescriptions were key factors influencing drug consumption patterns [44, 45].

Nationally and regionally, celecoxib sales were higher than etoricoxib, which also presented more structural breaks, indicating changes in sales patterns of this medicine. Celecoxib is indicated for treating various clinical conditions, such as rheumatoid arthritis, osteoarthritis, dysmenorrhea, and acute pain [46]. Etoricoxib is used primarily for acute pain relief and is also used in the chronic treatment of signs and symptoms associated with osteoarthritis and rheumatoid arthritis [47]. A clinical trial conducted in Korea revealed that etoricoxib was not inferior to celecoxib in treating osteoarthitis [48], but the safety profile of etoricoxib seems to be more concerning than that of celecoxib. A multinational assessment of 23 504 participants indicated that etoricoxib increased both systolic and diastolic blood pressures, compared with diclofenac [49]. A systematic review conducted in 2017 included 28 studies and revealed that the gastrointestinal risk of etoricoxib could be up to twice that of rofecoxib and almost triple that of celecoxib [10]. Such discrepancies may favor celecoxib preference over its counterparts and may explain its higher consumption observed in this study.

## 5 Conclusion

Coxib consumption in Brazil increased from 2014 to 2021, with celecoxib showing the most consistent growth. Structural breaks were observed in some regions and nationally for etoricoxib, indicating shifts in consumption patterns. Taking population growth into account, coxibs sales still showed a marked increase, suggesting a persistent demand for these drugs. Future investigations incorporating clinical data and population aging trends may provide further evidence on coxib consumption patterns in Brazil.

### 5.1 Plain Language Summary

This study assessed coxibs sales trends in Brazil from 2014 to 2021. We analyzed nationwide sales and measured how much of these drugs were used in different regions (North, Northeast, South, Southeast, and Midwest). Celecoxib and etoricoxib sales increased during this period. Celecoxib sales more than doubled, particularly in the Northeast region. Etoricoxib sales also increased, especially in the Midwest. Northern Brazil showed the lowest consumption. This increase in coxibs sales suggests that measures taken a decade before to ensure rational use of these drugs may not have worked as intended. This study used data from the Brazilian National Controlled Products Management System (*Sistema Nacional de Gerenciamento de Produtos Controlados*, SNGPC) on drug sales in Brazilian drugstores, but lacked patient age, sex, and prescription reasons. The results took into consideration population growth—but not aging—and highlight the need to assess the reasons for increasing the use of coxibs in Brazil.

## Ethics Statement

This research was based on public and de‐identified databases and thus dispensed ethical appreciation according to Brazilian regulation.

## Conflicts of Interest

The authors declare no conflicts of interest.
